# Integrin-Specific Control of Focal Adhesion Kinase and RhoA Regulates Membrane Protrusion and Invasion

**DOI:** 10.1371/journal.pone.0074659

**Published:** 2013-09-09

**Authors:** Patricia Costa, Tim M. E. Scales, Johanna Ivaska, Maddy Parsons

**Affiliations:** 1 Randall Division of Cell and Molecular Biophysics, King’s College London, London, United Kingdom; 2 Medical Biotechnology, VTT Technical Research Centre of Finland, Turku, Finland; 3 Centre for Biotechnology, University of Turku, Turku, Finland; Stony Brook University, United States of America

## Abstract

Cell invasion through extracellular matrix (ECM) is a hallmark of the metastatic cascade. Cancer cells require adhesion to surrounding tissues for efficient migration to occur, which is mediated through the integrin family of receptors. Alterations in expression levels of β1 and β3 integrins have previously been reported in a number of human cancers. However, whether there are specific roles for these ubiquitous receptors in mediating cell invasion remains unclear. Here we demonstrate that loss of β1 but not β3 integrins leads to increased spread cell area and focal adhesion number in cells on 2D immobilized fibronectin. Increased adhesion numbers in β1 knockdown cells correlated with decreased cell migration on 2D surfaces. Conversely, cells depleted of β1 integrins showed increased migration speed on 3D cell-derived matrix as well as in 3D organotypic cultures and inverted invasion assays. This increased invasive potential was also seen in cells lacking β3 integrin but only in 3D cultures containing fibroblasts. Mechanistically, in situ analysis using FRET biosensors revealed that enhanced invasion in cells lacking β1 integrins was directly coupled with reduced activation of focal adhesion kinase (FAK) and the small GTPase RhoA resulting in formation of enhanced dynamic protrusions and increased invasion. These reductions in FAK-RhoA signal activationwere not detected in β3 knockdown cells under the same conditions. This data demonstrates a specific role for β1 integrins in the modulation of a FAK-RhoA-actomyosin signaling axis to regulate cell invasion through complex ECM environments.

## Introduction

Cell adhesion is an essential requirement for normal embryonic development, adult homeostasis, and immune functions. Cancer cell invasion requires adhesion, proteolysis of the ECM components and migration. Migration involves cell polarization, protrusion and adhesion formation, actin polymerization and assembly and adhesion disassembly at the rear of the cell.

Cell:extracellular matrix (ECM) adhesion is mediated by integrins. Integrins are a family of 24 ubiquitous heterodimeric transmembrane receptors proteins and have been widely implicated as key regulators of cell migration and invasion [1]. Members of the family of β1 and β3 integrins have been shown to localize to focal adhesions (FA) and be capable of recruiting classical FA proteins such as paxillin, vinculin and focal adhesion kinase (FAK) to focal contact sites in many cell types [2]. Similarly, both receptors have been shown to trigger downstream signaling to members of the Rho family of small GTPases that are key molecular switches in the regulation of actin cytoskeletal assembly and cell migration [3]. However, despite many shared features there are also clear differences between these receptors in terms of function as exemplified from the study of the knockout mouse phenotypes. β1 null mice have a block in pre-implantation development and the embryo fails to gastrulate, whereas β3 integrin knockout mice are viable and fertile but have impaired platelet aggregation and thrombotic dysfunction [4], [5]. These distinct phenotypes demonstrate different, non-compensatory roles for these integrins during development and point towards potential distinct signaling pathways downstream of each receptor in specific *in vivo* contexts.

β1 and β3 integrins are both highly expressed in most invasive tumor cells, share common ligands (such a fibronectin) and intracellular binding partners (such as talin) and as such, similar roles have been attributed to these integrins during the invasion process. Previous studies have reported a role for β1 integrin in tumor cell growth [6], [7]. However, very few studies have previously analyzed the role for each integrin in the invasive process in the same cell type in parallel *in vitro* and *in vivo*. Furthermore, these studies have not been carried out in more physiologically representative 3-dimensional (3D) matrix environments that might better reflect the tumor microenvironment. This is important because recent studies have demonstrated that signals from the microenvironment, such as growth factors and ECM composition or structure, can also act as important regulatory cues for integrin-mediated signaling [8], [9], [10], [11]. In addition to crosstalk with growth factors, integrins have previously been reported to regulate positioning and activation of the matrix metalloproteinase (MMP) family of ECM proteases [12]. MMP’s play a key role in the control of specific local ECM degradation and biochemical complexes between both β1 and β3 integrin families have previously been reported in a range of cell types [13], [14], [15], [16], [17]. Integrins are therefore a potential key nodal point at which growth factor, ECM and protease signaling can converge. Thus, dissecting out integrin-specific signals and crosstalk with environmental cues potentially holds the key to understanding how each integrin contributes to the invasive process within differing ECM contexts and provide targets for therapeutic intervention.

In the present study we aimed to determine whether these integrin β receptor families have distinct or overlapping roles in controlling breast carcinoma cell adhesion and invasion. Our data demonstrates that individually silencing β1 or β3 integrins in human breast carcinoma cells results in an enhancement of invasion in both cases. We further show that β1 knockdown cells, but not those lacking β3, have lower active FAK and RhoA that leads to enhanced protrusion and invasion. This data demonstrates a novel mechanism by which specific β1 integrins can exert control over the actin cytoskeleton in response to specific extracellular cues and reveals an unexpected role for this receptor family in negatively regulating invasion in a context-specific manner.

## Results and Discussion

### β1 and β3 integrins differentially regulate cell morphology and adhesion formation

**Figure 1:**
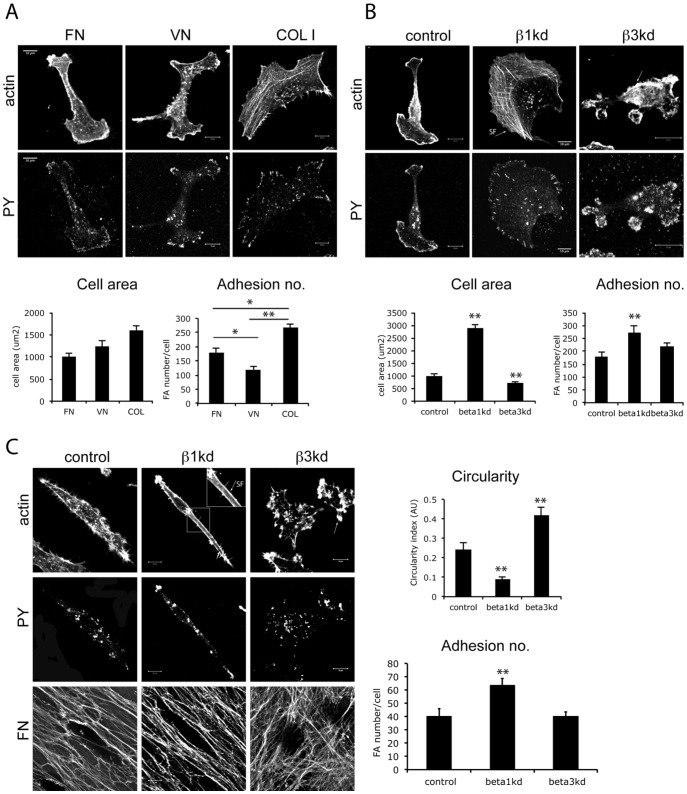
Differential control of actin and adhesion assembly by β1 and β3 integrins. (A) Example confocal images of control cells plated on fibronectin (FN), vitronectin (VN) or collagen I (COLI) fixed and stained with phallodin-Alexa488 and anti-phosphotyrosine (PY) antibody-Alexa 568. Scale bars 10μm. Graphs show quantification of cell area and mean number of focal adhesion (FA) per cell for each ECM. Bars are mean values +/−SEM., n  =  at least 75 cells for each over 3 independent experiments. (B) Example confocal images of β1 and β3kd cells plated on fibronectin (FN), fixed and stained with phalloidin-Alexa488 and anti- PY –Alexa 568 as general marker of focal adhesions. Arrows show stress fibers (SF) and actin ruffles in β1 and β3 kd cells, respectively. Graphs show quantification of cell area and mean number of focal adhesions per cell. Bars are mean values +/−SEM., n  =  at least 75 cells for each over 3 independent experiments. (C) Example confocal images of cells plated in cell-derived matrices (CDM). Top panels phalloidin-Alexa488; middle panels cells stained with anti-phosphotyrosine (PY)-Alexa568 as general marker of focal adhesions; bottom panels anti-fibronectin-Cy5. Arrows show stress fibers (SF) and actin protrusions in β1 and β3 kd cells, respectively. Graphs show quantification of cell circularity index (where 0 = elongated and 1 = rounded) and mean number of focal adhesions per cell. Bars are mean values +/−SEM., n  =  at least 20 cells for each over 3 independent experiments. *  =  p<0.01; **  = p<0.05 throughout compared to equivalent control values.

We first set out to determine whether β1 and β3 integrins could play similar roles in controlling cell morphology. We chose MDA MB 231 human breast carcinoma cells as a model system initially as these cells express high levels of β1 and β3 integrins and are known to be highly invasive both *in vitro* and *in vivo*. MDA MB 231 cells were plated on purified ECM ligands coated onto glass coverslips in serum-free media. Different ECM ligands were used to engage specific integrins: collagen (COLI; to engage β1), vitronectin (VN; to engage β3) or fibronectin (FN, to engage β1 and β3). Confocal imaging of fixed cells stained for F-actin and phosphotyrosine (PY; a general marker of focal adhesion signaling proteins that are heavily tyrosine phosphorylated) showed that these cells exhibited distinct morphology and adhesion assembly profiles when plated on purified ligands immobilized on coverslips (Figure 1A). On COLI (β1 ligand) cells showed the highest spread cell area and increased focal adhesion number, whereas on the β3 ligand VN, cells had fewer FA when compared to cells plated on the shared integrin ligand FN (Figure 1A). This suggested specific roles for these integrins in controlling cell morphology and actin cytoskeletal architecture. In order to test our hypothesis that β1 and β3 integrins trigger distinct morphological phenotypes, we generated two cell lines stably expressing shRNA to specifically knockdown (kd) each integrin (β1kd and β3kd respectively). Integrin kd cell lines were used interchangeably or in parallel throughout the study with very similar results (Figures S1A, B; 3B; 5). Each showed ∼95% knockdown efficiency compared to control shRNA-expressing cells and β1kd resulted in a decrease in expression levels of α2, α3 and α5 integrin partner subunits as has previously described to occur in other integrin null cell types; [4], [18], [19] but no change in total β3 integrin levels (Figure S1A, B). Similarly, depletion of β3 resulted in decreased levels of the β3 partner αv, but no change in β1 levels (Figure S1A, B). Silencing β1 or β3 subunits significantly reduced cell adhesion to collagen and vitronectin respectively as expected (Figure S1C). However, adhesion to fibronectin was modestly increased in both cell lines suggesting each integrin can compensate for the loss of the other in ligand-binding, as has been show previously in β-integrin knockout fibroblasts [19], [20]. Additionally, silencing β1 or β3 subunits resulted in significant changes in cell spreading and focal adhesion assembly on the shared ligand FN (Figure 1B, Figure S2). β1kd cells showed significant increase in cell area and FA number, whereas β3kd cells showed decreased cell area but without any change in FA number when plated on FN. Moreover, β1kd cells assembled more F-actin rich stress fibers whereas β3kd cells instead assembled peripheral F-actin-rich membrane ruffles (Figure 1B-arrows). These phenotypes were also evident in cells plated in 3D cell-derived matrices (CDM) mainly composed of fibrillar fibronectin and collagen, but rather than showing larger spread area, β1kd cells were instead highly elongated, branched along ECM fibers and with more adhesions and stress fibers along the length of the cell (Figure 1C-inset, arrow). Conversely, β3kd cells were more rounded with multiple short protrusions (Figure 1C-arrow). Further confocal microscopy analysis of β1 localization in β3kd cells and vice versa revealed distinct recruitment and activation of the remaining integrin in the absence of the other (Figure S2). This is in agreement with our adhesion data and further suggests possible trans-dominant action of each integrin over the other as we and other have described previously ([21], [22], [23], [24], resulting in one group of receptors suppressing activation of another. This suggests that β1 or β3 integrins can regulate cell morphology and adhesion assembly in distinct ways in cells within physiologically relevant 3D ECM environments.

### β1 integrins regulate cell migration and invasion

**Figure 2:**
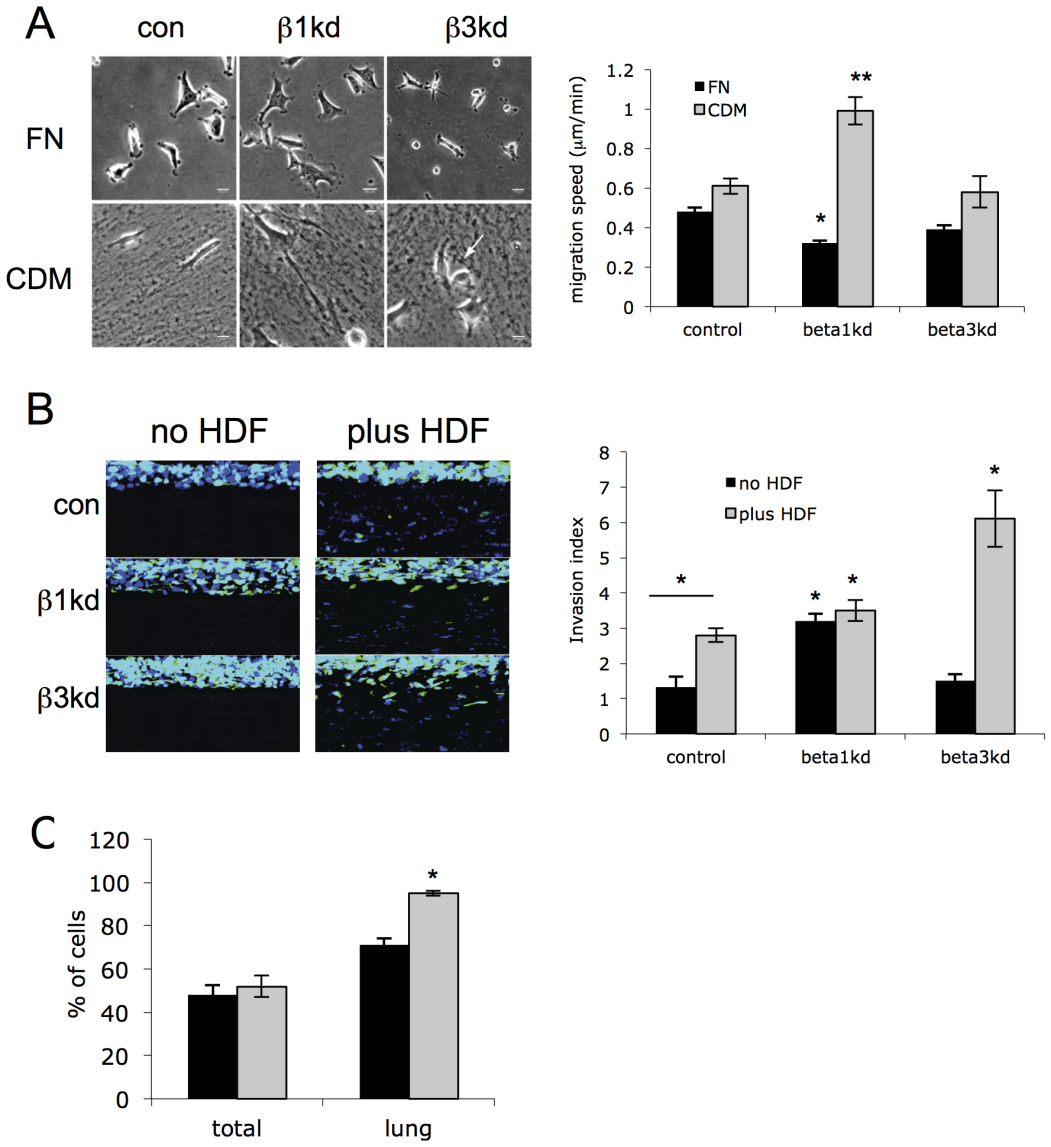
Silencing β3 integrins results in increased fibroblast-dependent cell invasion. (A) Example phase contrast images from time-lapse movies of specified cells plated on fibronectin or CDM. Graph shows quantification of migration speed from time-lapse movies of cells on specified ECM proteins. Bars are mean speed (μm/min) +/− SEM, n =  at least 110 cells over 3 independent experiments. (B) Example fluorescence confocal images of organotypic cultures either containing human dermal fibroblasts (HDF) or not. MDA MB 231 cells are represented in green (blue  =  DAPI). Scale bars are 50μm. Graph shows quantification of invasion of cells in organotypic cultures. Bars represent mean invasion index +/− SEM from at least 40 different images per cell type over 4 independent experiments. *  =  p<0.01 throughout compared to equivalent control values. (C) Analysis of metastasis of control or β1kd cells in nude mice. MDA-MB-231 cells were pre-labeled with fluorescent cell trackers and intravenously injected (5×10^5^ green and 5×10^5^ red cells together) into mice. After 48 hours the cells remaining in the vasculature were stained with mouse anti-human HLA-antibody for 5 min. Cells from one lung per mouse were isolated, stained with Alexa-647 secondary antibody and quantified based on fluorescence. Results are expressed as (mean±SEM) percentage of specified cells from all cells isolated (n = 10 mice; *, p = 0.05).

In order to determine whether the specific phenotypes of these cells led to altered cell motility, we analyzed the rate of migration in β1kd and β3kd cells on both 2D FN surfaces and in 3D CDM. Tracking of time-lapse movies and subsequent analysis revealed that compared to control cells, β1kd cell migration speed was reduced when plated on the shared ligand FN. However, migration speed was enhanced in β1kd cells plated in CDM (Figure 2A), in agreement with previous studies showing increased migration in cells derived from β1−/− mice plated in CDM [25]. Conversely, β3kd cells showed no changes in migration speed on FN and CDM, suggesting that β1 specific engagement with complex fibrillar ECM acts to slow cell migration speed. This suggests that chemical and topographical composition of 3D ECM can dramatically alter integrin-specific migration modes, and that changes in migratory modes in 3D ECM environments is mediated primarily through β1 integrins.

Stromal-epithelial interactions are becoming increasingly recognized as important players in controlling tumor growth and cell invasion [8], [26]. Stromal fibroblasts are responsible for the synthesis of growth and survival factors, angiogenic and immunological chemokines, and for the synthesis, deposition and remodeling of the structural components of the ECM as well as enzymes that control its turnover [27], [28]. Recent studies have shown that stromal fibroblasts can promote invasion either through release of soluble factors, ECM remodeling or direct cell interactions [8],[10],[26]. To determine whether the different roles we observed for β1 and β3 integrins in controlling migration also altered invasion in more complex ‘tissue-mimic’ environments, we seeded cells into ‘organotypic’ 3D ECM cultures either in the presence or absence of normal human fibroblasts [29], [30]. Cultures were grown for 10 days, followed by fixation and sectioning to permit quantification of invasion in each cell line (Figure S3A). Analysis demonstrated that β1kd cells showed enhanced invasion compared to control cells in cultures without fibroblasts, but that no further increase in invasive capability was seen in the presence of fibroblasts (Figure 2B). By contrast, β3kd cells exhibited similar invasive behavior to control cells in culture without fibroblasts, but exhibited a dramatic increase in invasion in the presence of fibroblasts (Figure 2B). Furthermore, we confirmed this finding in another human breast cancer cell line MDA MB 468 as well as a human melanoma-derived cell line (MDA MB 435) (Figures S3C, D) implying that these effects of β1 depletion are not specific to one cancer cell line. In order to determine whether β1kd cells also undergo constitutively enhanced invasion *in vivo*, these cells were also analyzed for their ability to colonize the lung in experimental cancer cell extravasation assays in nude mice. Fluorescently labeled control and β1kd cells (Figure S3E) were mixed 1:1 and injected into the tail vein and extravasation to the lung analyzed after 48 hours. In agreement with the two *in vitro* models of invasion, analysis demonstrated a significant increase in the percentage of β1kd cells in the lungs compared to controls (Figure 2C). These combined findings demonstrate that loss of either β1 or β3 integrins result in enhanced invasion *in vitro* and *in vivo* in response to different extracellular microenvironments. Previous studies have reported decreased proliferation and tumor growth *in vivo* in β1-depleted cells [6], [7]; however, the appearance of longer-term metastasis from these smaller tumors was not monitored. Moreover, analysis of metastatic invasion of cells from solid tumors would be difficult to interpret given the smaller size of the initial primary β1-silenced cell lesions. Hence it is possible that whilst β1kd cells show reduced growth, this may predispose cells to an increased chance of escaping the primary tumor and undergoing metastasis to distant sites.

### β1 integrins control matrix degradation in 2D environments

Previous reports have shown roles for integrins in mediating activation of the matrix metalloproteinase (MMP) family of ECM proteases. Integrins can form a complex with MMP’s and are proposed to act as membrane tethers for the inactive protease to promote highly localized sits of activation and ECM degradation [13], [14], [15], [16], [17]. In order to determine whether β1 or β3 knockdown cells control invasive cell behavior through modulation of MMP activation, we performed zymography analysis of conditioned media collected from each cell line. Data demonstrated no difference in activation, levels or localization of MMP9 or MT1MMP collagenases between cell lines suggesting that silencing these integrins does not primarily control invasion through altered global MMP activity (Figures S4A-C). To further analyze whether knockdown of either β1 or β3 integrin may alter cellular degradation of ECM, we plated cells on 2D fluorescently-labeled gelatin and measured degradation [31]. Despite not showing differences in MMP activation, data demonstrated that β1kd, but not β3kd cells showed a small but significant reduction in 2D gelatin degradation compared to control cells (Figures S4D, E). Given that β1kd cells show decreased migration and increased assembly of focal adhesion on FN, we postulate that this reduced mobility is likely to alter the ability of β1kd cells to degrade 2D matrix. Our data shows that knockdown of β1 integrins results in increased invasion of cells within 3D CDM, organotypic models or *in vivo* and therefore that 3D environments can dramatically switch cell phenotype. Importantly this suggests that measuring degradation on 2D surfaces does not necessarily reflect cell invasion ability within complex 3D fibrillar ECM environments.

### β1 integrin specific signaling suppresses protrusion formation and invasion through RhoA

**Figure 3:**
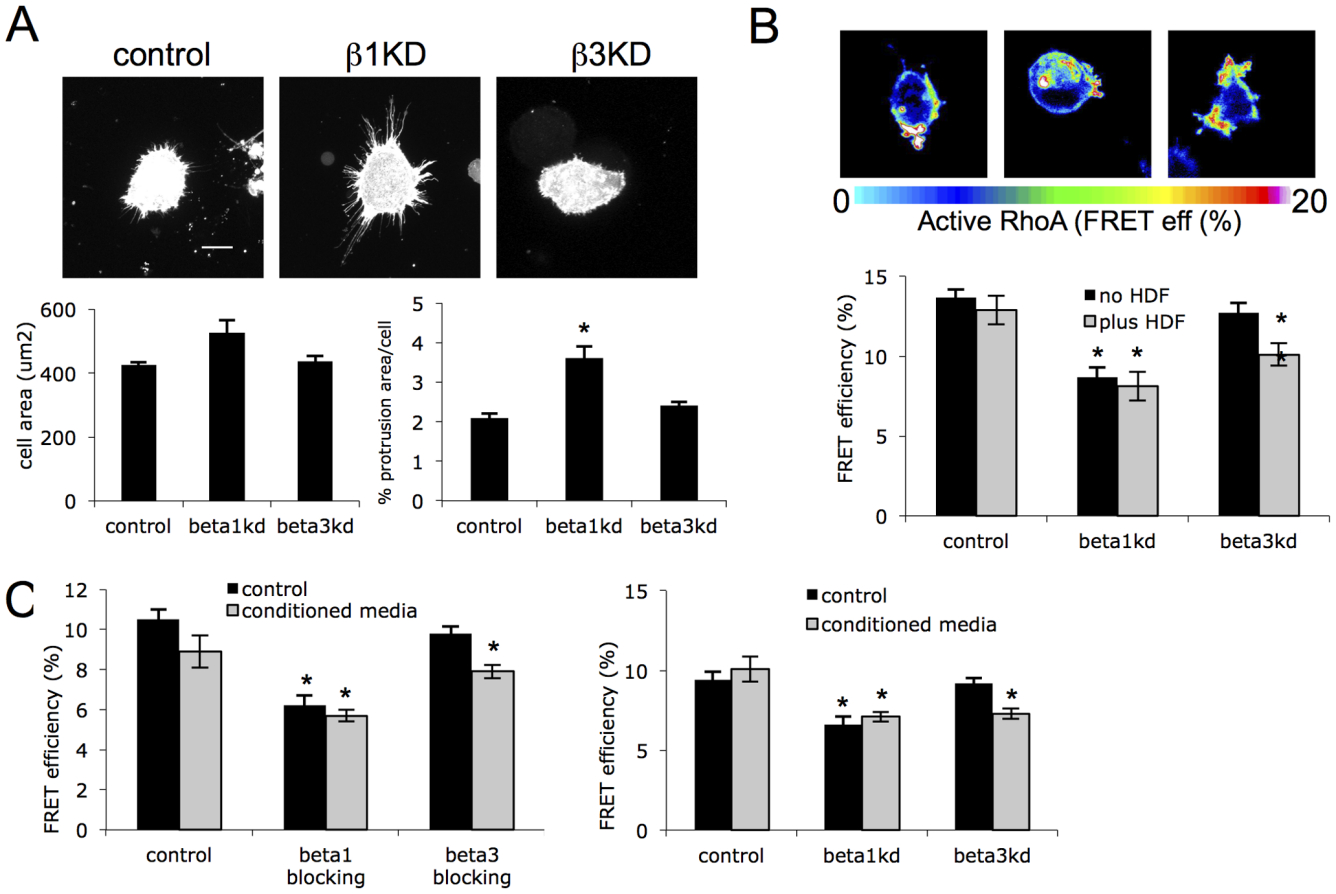
β1 and β3 integrins differentially contribute to RhoA activation during invasion. (A) Z-projections of >25 confocal z-stack images of specified cells expressing GFP-lifeact embedded in 3D ECM gels. Scale bar is 10 µm. Graphs show mean cell area and % of cell area occupied by membrane protrusions quantified from reconstructed confocal z-stack images of GFP-lifeact cells as shown. At least 35 cells quantified for each, error bars are SEM. * denotes p<0.01. (B) Example images and quantification of FRET analysis of RhoA activation in each cell type. Cells cultured in 3D gels either in presence or absence of human dermal fibroblasts (HDF). Bars show mean FRET efficiency (%) +/−SEM, n =  24 for each over 3 independent experiments. (D) Quantification of RhoA activation using analysis of RhoA FRET biosensor in control cells treated with control or integrin function blocking antibodies (left graph) or integrin knockdown cells plated in 3D gels in the presence of control media or conditioned media from human dermal fibroblasts (HDF). Bars are mean FRET efficiency +/−SEM, n = 30 cells over 3 independent experiments. * = p<0.01.

**Figure 4.**
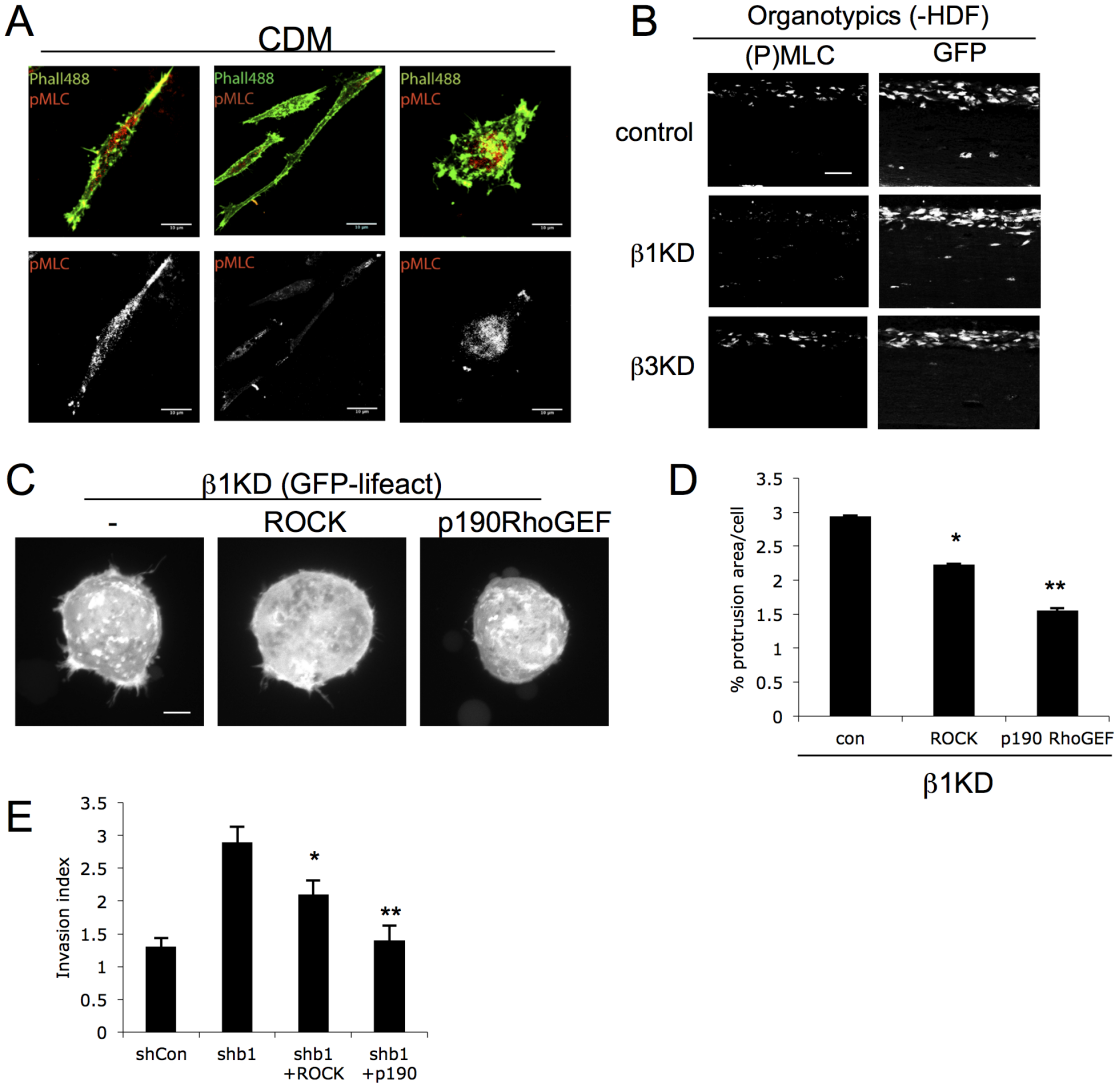
Fibroblast-dependent cell invasion is regulated by β1-dependent modulation of RhoA activity. (A) Example confocal images of cells plated in cell-derived matrices (CDM) and stained for phalloidin-Alexa488 (green) and (P)MLC-Alexa568 (red). Bottom panels show (P)MLC channel only. Scale bars are 10 µm. (B) Example confocal images of organotypic cultures stained with antibodies to (P)MLC (left panels). MDA MB 231 (GFP) cells are shown in right panels. Scale bars are 50 µm. (C) Example projected images of >15 confocal z-slices of control or knockdown cells expressing GFP-lifeact. Scale bars are 10 µm. (D) Quantification of protrusion area as a function of total cell area calculated from images as in (C). Bars represent mean % protrusion area per cell +/−SEM from 50 cells over 3 independent experiments. ** = p<0.01, * = p<0.005. (E) Quantification of invasion of shCon cells or β 1kd cells expressing ROCK or p190RhoGEF in organotypic assays in the absence of HDF (as in (B). Bars represent invasion index+/−SEM from 25 images over 2 independent experiments. ** = p<0.01, * = p<0.005.

In order to determine whether the enhanced constitutive invasion of β1kd cells in 3D ECM was coupled with altered cytoskeletal assembly (seen in cells on 2D ligands), the assembly of peripheral F-actin protrusions was quantified from confocal z-stack reconstructions of lifeact-GFP expressing cells embedded in 3D matrices. Control cells assembled a large number of F-actin rich protrusions that emanated from the entire surface of the cell (Figure 3A, B) and formed contacts with the extracellular matrix fibers in these 3D scaffolds as we have reported previously [32]. Analysis of 3D reconstructions in multiple cells revealed a significant increase in protrusion formation in β1kd but not β3kd cells compared to controls (Figure 3A, Figure S5). The consistently enhanced protrusions seen in these cells were not associated with increased cell rounding or cell death (data not shown) but rather the formation of F-actin rich protrusive structures. This finding agrees with the observed increased stress fiber assembly in these cells on FN and CDM (Figure 1B, C) and suggests that these receptors act distinctly in mediating F-actin assembly in 2D and 3D environments. The small GTPase RhoA is known to be an essential regulator of the actin cytoskeleton acting downstream of integrin engagement [33], [34], [35], [36]. As our data demonstrated integrin-specific changes in F-actin based protrusions in both 2D and 3D matrices, we sought to determine whether these phenotypes were coupled with changes in activation of RhoA. In order to visualize and quantify RhoA activation in intact cells in 3D ECM, we made use of the Raichu RhoA CFP/YFP FRET biosensor that has previously been shown by our lab and others to report directly on localized changes in active RhoA [37], [38]. Cells expressing the RhoA FRET biosensor were embedded in 3D ECM gels as before in the presence or absence of fibroblasts and activation measured in a series of Z-stacks using acceptor photobleaching FRET analysis. Analysis revealed that active RhoA levels negatively correlated with invasive phenotype in each cell line; β1kd cells exhibited constitutively lower levels of active RhoA, whereas β3kd cells showed a decrease in RhoA only in the presence of fibroblasts, compared to control cells (Figure 3B). Moreover, these altered levels of active RhoA in each cell line were recapitulated in control cells treated with integrin-specific blocking antibodies, confirming that ligand-engagement or activation of each integrin was required to elicit the observed changes in RhoA activity (Figure 3C). Furthermore, these changes in active RhoA were also seen in cells embedded in 3D ECM and treated with conditioned media from fibroblasts, suggesting that soluble factors released from stromal cells, rather than direct interactions between fibroblasts and tumor cells, contributed to enhanced RhoA activation (Figure 3C). Importantly, we did not detect significant changes in RhoA activation in integrin knockdown cells plated on 2D surfaces (Figure S6A) further supporting the idea that the ECM microenvironment and architecture plays a key role in dictating specific signaling downstream of each integrin. To determine whether the observed β1-dependent decrease in active RhoA also led to reduced actomyosin contractility, we analyzed levels and localization of phosphorylated myosin light chain (P-MLC), as a known downstream effector of RhoA in this pathway. Whilst no differences were seen by western blot analysis of total P-MLC levels in cells on 2D (Figure S6B), immunostaining of cells undergoing invasion into CDM or 3D matrices showed a clear reduction in P-MLC in β1kd cells (Figure 4A, B) suggesting that the lower RhoA activity in these cells results in lower levels of actomyosin contractility.

RhoA is known to play a key role in stabilizing the actin cytoskeleton and this in turn influences assembly and disassembly of F-actin based protrusions (for review see [3]). As our analysis demonstrated an inverse correlation between RhoA activity and protrusion formation/cell invasion, we reasoned that these observations could be functionally linked. To test this, we overexpressed an upstream activator of RhoA, p190RhoGEF that has also been previously shown to be important in integrin-mediated signaling [39], [40] or a downstream RhoA effector Rho kinase (ROCK) in β1kd cells and analyzed protrusion formation in cells within 3D matrices. Quantification revealed that high levels of P190RhoGEF was sufficient to fully restore protrusion assembly back to levels seen in control cells (Figures 4C, D). Expression of ROCK also significantly reduced protrusion formation but only by around 50% (Figure 4C, D) suggesting that additional RhoA effectors, such as mDia, may co-operate to regulate protrusion assembly in invasive cells as has been previously demonstrated [41]. Importantly, the reduction in protrusion formation seen in β1kd cells correlated with a significant decrease in invasion also measured in these cells (Figure 4E). These data combined demonstrate that β1 (but not β3) integrins act to suppress protrusion formation and invasion by maintaining high active RhoA levels and thus acting to induce downstream contractility.

### β1 integrins control activation levels of FAK and RhoA to suppress invasion

**Figure 5:**
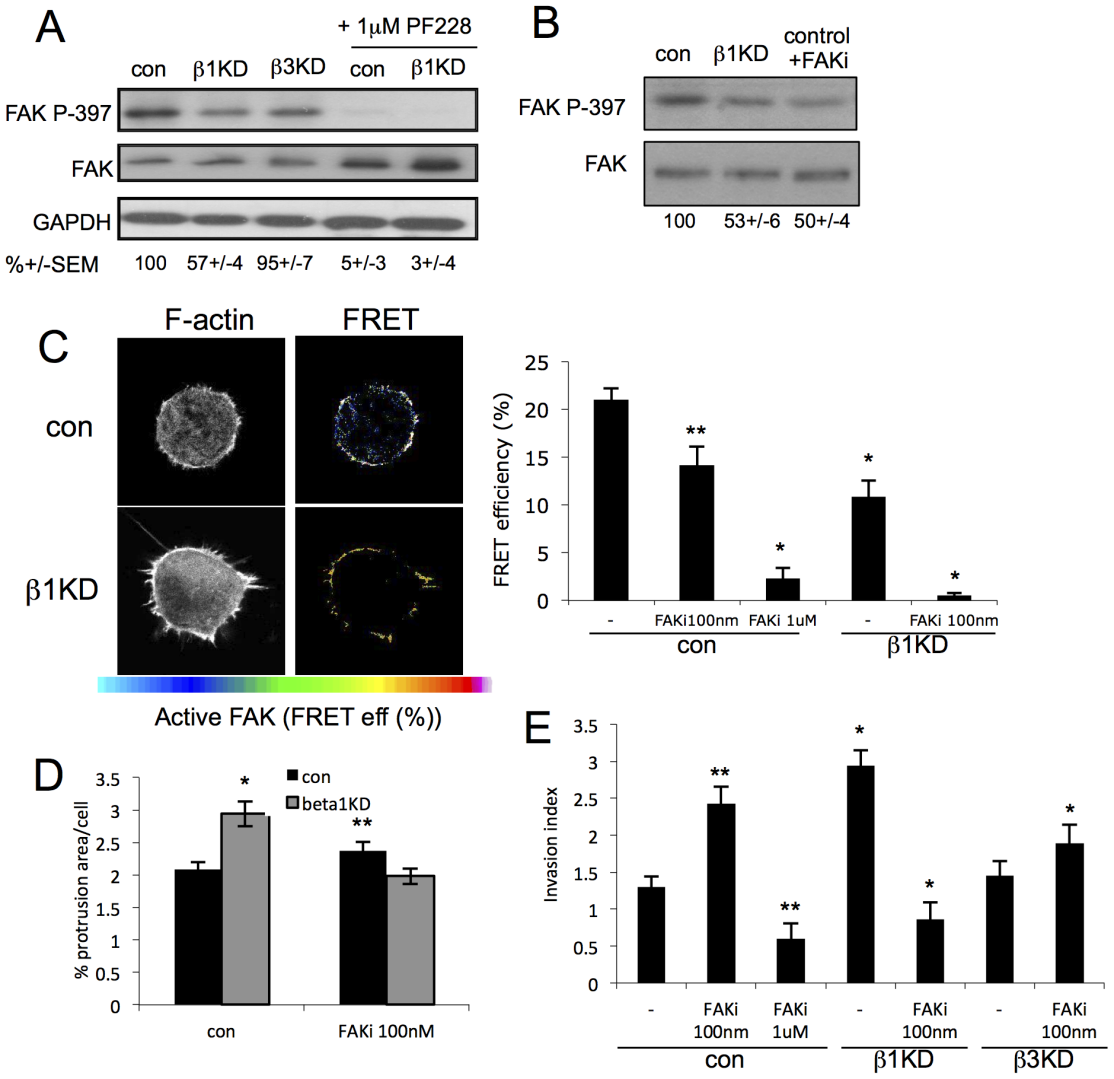
Enhanced invasion in β1-silenced cells is regulated by attenuated FAK activity. (A) Western blot of lysates from specified cells either untreated or treated with 1 µM PF228 (FAK inhibitor) for 2 hours. Blot is probed for active (P-397) or total FAK. GAPDH serves as a loading control. Numbers below represent average active FAK levels as a % of control as quantified by densitometry from 4 independent experiments +/−SEM. (B) Western blot of lysates from shCon or β1kd cells treated with vehicle control or PF-228 at 100 nM (FAKi) and probed for P-FAK (Y-397) or total FAK. (C) Example images of shCon or β1kd cells expressing FAK FERM FRET biosensor embedded in 3D gels. Images in left panel show F-actin (phalloidin) and right panels show FRET efficiency heatmaps according to pseudocolour scale bar indicated. Graph shows quantification of >30 cells per specified condition. Bars represent mean FRET efficiency+/−SEM across 5 independent experiments. ** = p<0.01, * = p<0.005. (D) Quantification of protrusion area/cell of control or β1kd cells expressing GFP-lifeact and embedded in 3D gels. Cells were treated with DMSO or PF228 at 100 nM prior to analysis. Bars represent mean+/−SEM of 45 cells each over 2 experiments. * = p<0.01 (E) Quantification of invasion of specified cells into 3D gels treated with DMSO (vehicle control) or PF-228 at specified concentrations. Bars represent mean+/−SEM or 35 images across 3 independent experiments. ** = p<0.01, * = p<0.05.

We next sought to identify the potential molecular mediators of β1-dependent protrusion assembly and invasion in these cells. Focal Adhesion Kinase (FAK) is a well-characterized component of many cell adhesion types and is one of the key mediators of integrin-dependent signaling controlling cancer cell invasion [42]. Recent studies have demonstrated that p190RhoGEF binds to, and is a substrate for, FAK and that FAK-dependent phosphorylation acts to control GEF activity [39], [43]. We therefore speculated that FAK could play a role in controlling active RhoA levels directly downstream of β1 integrins. FAK is autophosphorylated on Tyr-397 (p-Y397) and this is widely used as a biochemical reporter of activation of downstream targets. To determine whether activation of FAK was differentially regulated by β1 and β3 integrins, we first analyzed p-Y397 levels in control or integrin silenced cells plated in CDM as these cultures were amenable to biochemical analysis. Western blotting of cell lysates demonstrated a 43% reduction in active (p-Y397) FAK in β1kd cells, but no change in β3kd cells compared to controls (Figure 5A). Treatment of control or β1kd cells with a FAK-specific inhibitor (PF-573, 228, herein referred to as PF-228; [44]) confirmed specificity of the P-Y397 immunoreactive bands in these lysates and that both cell lines were susceptible to inhibition by this compound at 1 µM as has been shown previously in other cell lines (Figure 5A; [44]). Further analysis of control cells treated with lower concentrations of PF-228 demonstrated decreased p-Y397 FAK to levels seen in β1kd cells (Figure 5B) demonstrating that this compound could be used to fine-tune FAK activation in parental cells. Similar reductions in active FAK were observed in MDA MB 468 cells treated with 500 nM or 2.5 µM of PF-228 (Figure S7A). In order to determine whether FAK activity was also suppressed in β1kd cells undergoing invasion into 3D matrices, we made use of a recently developed FAK FERM FRET sensor [45] to analyze activation of this kinase *in situ* in intact cells. Acceptor photobleaching FRET analysis of cells in 3D confirmed significantly lower active FAK levels in β1kd cells compared to controls (Figure 5C). Moreover, FRET efficiencies in control cells treated with different concentrations of PF-228 demonstrated a clear decrease in active FAK in agreement with our biochemical data (Figure 5A-C). Interestingly, the active FAK species localized to the base of F-actin protrusions in both cell lines, but the attenuated active FAK in β1kd cells was more punctate and distributed along the larger protrusions (Figure 5C) suggesting loss of spatially regulated FAK activity in these cells.

Our observations of decreased active RhoA leading to increased protrusion assembly and invasion in β1kd cells prompted us to address whether attenuated FAK may also play a role in these phenotypes. To this end, we treated GFP-lifeact expressing control or β1kd cells within 3D matrices with 100 nM PF-228 and analyzed F-actin protrusion formation as before. Data revealed a significant increase in protrusion formation in control cells at this dose, further supporting our hypothesis that attenuating FAK activity can lead to formation of large protrusions (Figure 5D). This data would also suggest that bypassing the requirement for β1 integrin signaling by directly suppressing active FAK results in a similar morphological phenotype suggesting FAK is the primary pathway downstream of this integrin in mediating protrusion assembly or stability. Interestingly, using the same dose of PF-228 to inhibit the remaining active FAK in β1kd cells (100 nM) was sufficient to inhibit the enhanced protrusion formation, implying that FAK activity is required for this phenotype (Figure 5D). Finally, in order to determine whether the invasive capacity of cells correlated with FAK-dependent protrusion, we analyzed invasion in 3D matrices without fibroblasts in all cell lines with or without PF-228. Control cells treated with 1 µM PF-228 (that is sufficient to inhibit FAK activity; Figures 5A,C) resulted in a significant reduction in invasion (Figure 5E) in agreement with previous studies showing FAK activation is required for cell invasion [46], [47]. However, treatment of either control, or β3kd cells with a lower dose of PF-228 that reduces, but does not inhibit, FAK activity (Figures 5B,C) resulted in enhanced invasion in both cell lines (Figure 5E). Conversely, treatment of β1kd cells with 100 nM PF-228 instead resulted in inhibition of invasion, suggesting that silencing β1 integrin that results in decreased active FAK, acts to sensitize these cells to FAK inhibitors. A similar non-linear invasion response to an intermediate and high dose of the FAK inhibitor were also observed in MDA MB 468 cells (Figure S7B). The ability of each cell type to invade under these conditions also directly correlates with the assembly of actin-based protrusions (Figure 5D), further implying that the two phenotypes are coupled in a β1- and FAK-dependent manner. Thus our data support a model in which β1 integrins specifically control the balance of active FAK that in turn regulates RhoA-dependent actin-based protrusion assembly and cell invasion.

## Conclusions

Here we provide evidence that β1, but not β3 integrins, play a key role in controlling activation of FAK and RhoA to dictate local F-actin dynamics that contribute to cell invasion. Furthermore our data highlights the previously unrecognized importance of fine-tuning levels of active FAK in invading carcinoma cells. We propose that contrary to enhancing invasion, β1 integrin ligand engagement may in fact act as a ‘brake’ on invasion in certain cell types by activating FAK-RhoA signaling and suppressing or stabilizing dynamic invasive protrusions. This data has important implications for the design and dosing of therapies to prevent metastatic disease and suggests that dual inhibition of β1 integrins and FAK may be a powerful approach to inhibit both primary tumor growth and invasion.

## Materials and Methods

### Antibodies, constructs and reagents

Phalloidin and all Alexa-conjugated secondary antibodies were purchased from Invitrogen. Other antibodies were purchased from the following sources: Anti- β1 (clone 4B7), active- β1 (clone 12G10), anti-α2 (clone P1E6), anti- α3 (clone 29A3), anti-α5 and -αv, anti-MT1MMP (clone LEM2/15.8), anti-FN (rabbit) and anti-GAPDH were all from Millipore; anti- β3 (clone PM6/13, Serotec); anti-active β3 (clone CRC54, Abcam); anti-PY (clone 4G10, Upstate Biotechnology), anti- β5 (clone 4AK, Santa Cruz); anti-total and (P)18/19 MLC, anti-FAK and Y397-P FAK antibodies (# 3285 and 3283) and were from Cell Signaling Technology. ROCK-mRFP was a gift from Gareth Jones (King’s College London), RhoA Raichu biosensor was a gift from M. Matsuda (Kyoto University) and the FAK FERM biosensor was a gift from Gertrude Bunt (Max-Planck Institute of Experimental Medicine, Göttingen; [45]). PF-228 was purchased from Merck biosciences. GFP-N1 vector (used in organotypic experiments) was from Clontech. Lifeact-GFP was generated by inserting oligonucleotides encoding the lifeact peptide sequence [48] into the lentiviral backbone pHR’SIN-SEW (a gift from Adrian Thrasher (University College London; [49]).

### Cell culture and generation of stable knockdowns

MDA MB 231, 435 and 468 cells were purchased from ATCC and maintained at 37°C in DMEM or L-15 Medium containing 10% FCS, 1% penicillin/streptomycin and 1% glutamine (all from Sigma). The retroviral packaging cell line 293GPG were maintained in DMEM, 10% FCS, 1% penicillin/streptomycin and 1% glutamine, 2 µg/ml puromycin, 0.3 mg/ml G418 and 1 µg/ml tetracycline (Sigma). Primary human dermal fibroblasts were purchased from TCS Cellworks and maintained in HDF basal media plus supplements. Primary cells were only used between passages 1 and 15. Non-targeting control, β1 and β3 integrin shRNA retroviral clones in pSM2 backbone were purchased from Open Biosystems. Targets sequences for each shRNA was as follows: β1(1): CCACAGACATTTACATTAAA; β1(2): CAAATTGTCAGAAGGAGTAA; β3(1): GCCAGATGATTCGAAGAATT; β3(2): CAGGCATTGTCCAGCCTAAT. Retrovirus was generated for each by transfecting 293GPG cells with Fugene6 (Roche) for 24 hours followed by collection of virus-containing supernatant in Optimem (Gibco). MDA MB 231 cells were infected with resultant filtered virus plus 4 µg/ml polybrene (Sigma) for 24 hours followed by selection in 1 µg/ml puromycin. All cells were routinely checked for knockdown efficiency by western blotting. Cell-derived matrix (CDM) was prepared as described previously using primary human dermal fibroblasts [21].

### Western blotting and zymography

Cells were lysed in RIPA buffer (10 mM Tris [pH7.4], 150 mM NaCl, 1 mM EDTA, 1% Triton X-100, 1% sodium deoxycholate, 10 µM sodium fluoride, 1 µM okadaic acid with protease inhibitor complex (Calbiochem)). Cells were centrifuged at 13,000rpm for 5 min and lysate protein concentration was determined with the BCA assay (Pierce). Protein was loaded onto polyacrylamide gels and transferred to PVDF membranes. Membranes were blocked in TBS-T and 5% non-fat milk and incubated with specified primary antibodies in TBS-T overnight at 4°C. The immunoblots were washed in TBS-T and incubated for 2 hours with HRP-conjugated secondary antibody. Membranes were then washed with TBS-T and visualized with ECL substrate reagent (Pierce). For zymography analysis of MMP activation, MDA MB 231 cells were seeded at a density of 3×10^5^ cells per well onto 6 well plates coated with either 10 µg/ml FN, 50 µg/ml COL I or, 10 µg/ml VN in serum-free media (SFM). After 4h, media was replaced by 1 ml SFM. Cells were incubated for 24h at 37°C in a 5% CO_2_ atmosphere. Conditioned medium was collected and incubated with non-reducing sample buffer at 37°C for 15 min before loading onto the gel. Samples were subjected to electrophoresis in 10% polyacrylamide/0.05% gelatin gels. SDS was removed by washing the gels in 2.5% Triton X-100. Gels were incubated in 50 mM Tris-HCl (pH 8), 5 mM CaCl_2_ and 0.02% NaN_3_ for 3-24h and stained with 0.5% Coomasie blue R-250. For each sample whole cells lysates were prepared with sample buffer and blot for GAPDH as loading control. Quantification of pixels intensity was performed using Image J Software.

### Adhesion assays

MDA MB 231 cells were seeded at a density of 3×10^4^ cells/ml onto 24 well plates coated with either 10 µg/ml FN, 50 µg/ml COL I or, 10 µg/ml VN in SFM. Cells were allowed to adhere for 2h, wells were then washed with PBS and adherent cells were trypsinized and collected. Collected cells were counted with a hemocytometer and total number of cells per well was calculated.

### Time-lapse microscopy and migration assays

Phase contrast time-lapse imaging of cells as performed on a Zeiss Axio100 microscope equipped with Sensicam CCD camera (PCO Cooke), motorised stage (Ludl) and excitation/emission filters (Chroma) and filter wheels (Ludl). Images were acquired using a 10x phase objective. Random migration was performed on cells plated on 12-well tissue culture plates. Images were acquired, taking a frame every 10 min for 16 hours using IQ acquisition software (Andor). Subsequently all cells in the acquired time-lapse sequences were tracked using Andor Bioimaging Tracking. Tracking resulted in the generation of a sequence of position coordinates relating to each cell in each frame, motion analysis was then performed on these sequences using Mathematica 6 notebooks (Wolfram).

### Preparation of 3D gels and organotypic cultures

For analysis of cells in 3D, extra cellular matrix were prepared on ice as follows. Matrigel or Type I Rat Tail Collagen (both from BD Biosciences) were diluted to concentrations of 5 mg/ml or 2.5 mg/ml respectively in Optimem containing 20 mM HEPES and 10% (v/v) foetal bovine serum. Collagen matrix preparations also contained 0.3% (w/v) sodium bicarbonate. Matrices were either seeded with cells and transferred to LabTek 8-well imaging chambers, or were pipetted on top of cells previously plated in imaging chambers, before being allowed to polymerize at 37°C for 20 mins. Polymerised matrices were covered with 10% Foetal Bovine Serum in Optimem. Organotypic cultures were prepared as described previously (Ramsay et al, 2007). Primary human dermal fibroblasts (obtained from TCS Cellworks) were used where specified.

### Imaging and analysis of cells in 3D ECM

Cells and 3D ECM were fixed with 3.6% formaldehyde in PBS for 5 hr at 37°C, followed by several washes with PBS. As required cells were stained with phalloidin-568 by permeabilisation with 0.1% Triton TX-100 in PBS for 1 hr at room temperature, incubation with 1/200 dilution of phalloidin-568 in PBS for 4 hr (room temperature) and finally multiple PBS washes. Imaging of cells in 3D was performed using a Nikon A1R inverted confocal microscope using Plan Apo VC 60x Oil 1.4NA or Plan Fluor 40x Oil 1.3NA objective lenses. Image capture, analysis and 3D reconstructions were performed using NIS Elements software (Nikon). To measure invasion of cells into ECM, MDA-MB-231 cells stably expressing GFP-Lifeact were plated in LabTek 8-well chamber slides (2.5×10^3^ cells/well). After 24 hr 400 µl of matrix, as detailed above, was added on top of the cells. 24 hr post addition of matrix the cells were imaged on a Nikon A1R system as previously described using the 40x objective. For each condition 10 fields were imaged to a depth of 100 µm from the bottom of the well using z-steps of 0.75 µm. Invasion was quantified using NIS Elements software as follows. Maximum intensity x, z-projections were divided into a stack of regions of interest (ROIs) starting at the bottom of the well, each of 10 µm depth and equal to the width of the x, z-projection (318 µm). Cells were then thresholded and the area of each ROI covered by cells recorded. To correct for differing numbers of cells in each field the total area of cells in each x,z-projection was determined and then the percentage of this total cell area in each 10 µm ROI slice calculated. The degree of invasion was then expressed as an ‘invasion ratio’ calculated by dividing the percentage of total cell area that had invaded further than 30 µm by the area of cells that had not invaded past 30 µm. The distance of 30 µm was chosen as it was determined to be the distance at which cells that had extended any amount of their area past this point had to have completely detached from the bottom of the well and were therefore considered to be invading into the matrix. Invasion was quantified from three separate experiments in which 10 randomly chosen fields of view were analyzed per condition.

### Gelatin Degradation Assay

Fluorescent gelatin-coated cover slips were prepared as described [50]. Briefly, coverslips were coated with thin layers of rhodamine- conjugated gelatin (Sigma-Aldrich), cross-linked with 0.5% glutaraldehyde for 15 min and incubated for 3 min at room temperature with 5 mg/ml NaBH4. After three washes with PBS and 10 min incubation in 70% ethanol, coverslips were quenched with sodium borohydride (5mg/ml) for 1h at 37°C. Cells were seeded on gelatin-coated coverslips at a density of 1.5×10^5^ cells per well in complete DMEM. After 6h cells were fixed in 4% PFA. Phalloidin-488 was used to visualize F-actin. Analysis was performed on a Nikon A1R confocal microscope equipped with 60x/1.40 oil DIC Plan Fluor immersion objective. Quantification of total degradation was performed with Image J software.

### FRET analysis

Plasmid DNA encoding the CFP/YFP tagged versions of Raichu RhoA biosensor FRET probe or FAK FERM sensor were transfected into cells as specified. Nikon A1R inverted confocal microscope using Plan Apo VC 60x Oil 1.4NA or Plan Fluor 40x Oil 1.3NA objective lenses. Image capture, analysis and 3D reconstructions were performed using NIS Elements software (Nikon) as previously described [38], [51]. Briefly, the CFP and YFP channels were excited using the 440 nm diode and the 514nm argon lasers respectively. The two emission channels were split using a 545 nm dichroic mirror, which was followed by a 475−525 nm bandpass filter for CFP and a 530 nm longpass filter for YFP. Time-lapse mode was used to collect one pre-bleach image for each channel followed by bleaching with 50 iterations of the 514 nm argon laser line at maximum power (to bleach YFP). A second post-bleach image was then collected for each channel. Pre- and post-bleach CFP and YFP images were then imported into ImageJ for processing.

### *In vivo* experimental metastasis:

The animal work was carried out under license number ESAVI/7522/04.10.03/2012. Permissions for animal experiments are applied from the National Animal Experiment Board of Finland as required by the Finnish Act on Animal Experimentation. Mice are housed at the animal facilities at the University of Helsinki and the University of Turku under the conditions recommended by the European Convention for the Protection of Vertebrate Animals Used for Experimental and Other Scientific Purposes. Discomfort, distress, and injury are limited to unavoidable within the context of scientifically sound research. Trained veterinarians monitor animal welfare and handling, and the control and prevention of disease. Additional veterinary staff and veterinary technicians provide a complete and comprehensive program of diagnostics, preventive and clinical animal medicine and husbandry. Anesthesia is given for any potentially painful procedures. The method used here was as described in detail in [52]. Female athymic nude mice (Hsd:Athymic Nude-nu; Harlan Scandinavia, Allerod, Denmark), aged between 4−6 weeks, were used for the xenograft studies. All of the experimental procedures were approved by the local ethical committees. Mice were anesthetized with ketamine (Pfizer) and xylazine (Bayer). Transiently siRNA silenced (72h) MDA-MB-231 cells were stained with live cell dyes (Allstars-negative red, CMTPX; siITGB1 green, Qiagen siRNA 2142604, CMFDA: Invitrogen) according to manufacturer’s instructions. Cells were harvested, suspended in 50 µl PBS (5×10^5^ each), mixed and injected (control and siITGB1) into lateral tail vein of mice (n = 10). 48 h post-injection the mice were injected with mouse anti-human HLA antibody (Hb116; [53]) 3mg/ml for 5 min (to distinguish between human cells adhering to the vessel wall from those that have extravasated inside the tissue). Next the mice were anesthetized and the pulmonary vasculature was perfused with PBS through the right ventricle (2 min) and blood was allowed to escape by a small incision in the left atrium. Animals were sacrificed and cells were harvested from one lung per animal with collagenase XI treatment for 1 h +37 °C, washed with PBS, and cell number analyzed based on fluorescence using ScanR automated microscope.

### Statistical analysis

Experimental and analysis n numbers are stated in the corresponding figure legends. Statistical testing between datatsets was carried out using Student’s T-Test or ANOVA analysis where appropriate. Differences of p<0.05 or below were considered statistically significant and annotated on the figures accordingly.

## Supporting Information

Figure S1 **Generation of knockdown cell lines.** (A) Western blots of lysates from control (con) or β1, β3 knockdown (kd) cell lines probed for β1, β3 or GAPDH (loading control). Two different clones for each integrin are shown. (B) Western blots of lysates from integrin knockdown cells probed for specified integrin subunits. (C) Adhesion of each cell line to purified ECM proteins as specified. Cells were left to adhere in serum free media for 2 hours and remaining adherent cells counted. Data is presented as relative adhesion of β1kd or β3kd cells compared to shCon cells on same ECM protein. Data is pooled from 3 independent experiments, error bars are SEM. * = p<0.001.
(TIFF)

Figure S2 **Altered integrin activation in knockdown cells.** (A) Example confocal images of cells plated on fibronectin (FN), fixed and stained for specified integrins (either active or total). (B) As in (A) but cells plated on vitronectin (VN). (C) As in (A) but cells plated on Collagen I (COLI). (D) As in (A) but cells plated in cell-derived matrices (CDM). Scale bars 10 µm.
(TIFF)

Figure S3 **Integrin dependent invasion of human breast carcinoma cells.** (A) Example H&E stained sections of organotypic cultures using MDA MB 231 cells with or without fibroblasts. (B) cells expressing control shRNA or β1-specific shRNA (clone#2) in the absence of fibroblasts. (C, D) Quantification of cell invasion in 3D ECM organotypic model using MDA MB 435 (C) or MDA MB 468 (D) in the presence or absence of fibroblasts (HDF). (E) FACS analysis of β1-integrin levels on control and knockdown cells from the same experiments used for injection in mice for analysis of lung extravasation (Figure 2C). Bars are mean +/−SEM pooled from at least two independent experiments, each performed in triplicate.
(TIFF)

Figure S4 **Integrin knockdown results in increased cell protrusion in 3D matrices.** (A)Example projected images of >10 confocal z-slices of control or β1 integrin (clone#2) knockdown cells expressing GFP-lifeact. Scale bars are 10 µm. (B) Quantification of protrusion area as a function of total cell area calculated from images as in Fig 4. Bars represent mean % protrusion area per cell +/−SEM from 30 cells over 3 independent experiments. * = p<0.01.
(TIFF)

Figure S5 **Silencing** β**1 integrins does not alter MMP levels or activation but decreases 2D gelatin degradation**. (A) Example images of lysates from each cell line analysed on zymography gels for active MMP9 levels. Western blot of GAPDH also shown as loading control. Graph shows quantification of active MMP9 levels from zymography experiments. Values are from densitometry analysis from 4 independent experiments for each normalised for loading (from western blot analysis of total MMP9 for each experiment). Bars are mean+/− SEM. (B) Western blots analysis of total MT1-MMP levels in cell lysates. GAPDH is a loading control (C) Example confocal images of cells plated in cell-derived matrices and stained for MT1MMP or MMP9. Arrows show localised recruitment of MMP. (D) Example images from gelatin degradation assay. Cells plated on TRITC-gelatin (red, left panels, black and white in right panels), fixed and stained with phalloidin-Alexa488 (green). Arrows show area of gelatin degradation seen as black dots. (E) Quantification of degradation area normalised for total cell area (presented as µm^2^). Bars are average area +/−SEM. * = p<0.01 compared to control.
(TIFF)

Figure S6 **RhoA activation in 3D gels is integrin-dependent**. (A) Quantification of RhoA activation using analysis of RhoA FRET in cells plated on 2D glass coverslips. Bars are mean FRET efficiency +/−SEM, n =  18 cells over 3 independent experiments. (B) Representative blots of lysates from control or integrin silenced cells analysed by western blotting for levels of total or phospho (Ser18/Thr19)-myosin light chain (MLC). GAPDH serves as a loading control. Experiment performed 5 times with similar results.
(TIFF)

Figure S7 **Reduced active FAK leads to increased invasion in MDA MB 468 cells.** (A) Western blots of lysates from MDA MB 468 cells treated with vehicle control (con; DMSO), 50nM or 2.5mM of PF-228, lysed and probed for P-Y397 FAK or total FAK. Experiment was performed 3 times with similar results. (B) Quantification of invasion of MDA MB 468 cells into 3D gels treated with DMSO (control) or PF-228 (FAKi) at specified concentrations. Bars represent mean+/−SEM of 18 images across 3 independent experiments. * = p<0.05.
(TIFF)
