# Supplementary figures and images for: Integrin-Specific Control of Focal Adhesion Kinase and RhoA Regulates Membrane Protrusion and Invasion
Source: PLoS One. 2013 Sep 9;8(9):e74659. doi: 10.1371/journal.pone.0074659 (PMC3767638; doi:10.1371/journal.pone.0074659)

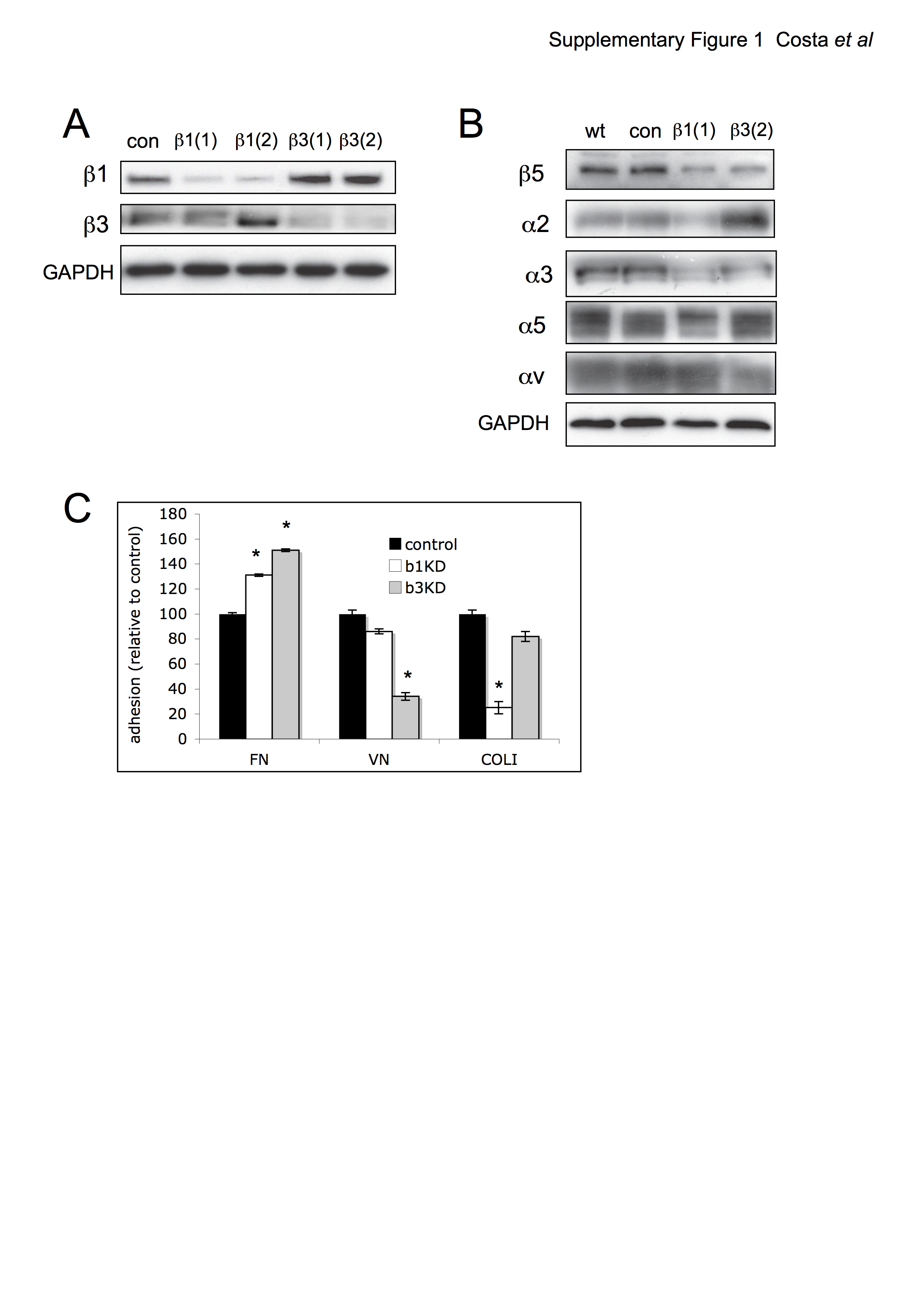

Supplement: Figure S1 — Generation of knockdown cell lines. (A) Western blots of lysates from control (con) or β1, β3 knockdown (kd) cell lines probed for β1, β3 or GAPDH (loading control). Two different clones for each integrin are shown. (B) Western blots of lysates from integrin knockdown cells probed for specified integrin subunits. (C) Adhesion of each cell line to purified ECM proteins as specified. Cells were left to adhere in serum free media for 2 hours and remaining adherent cells counted. Data is presented as relative adhesion of β1kd or β3kd cells compared to shCon cells on same ECM protein. Data is pooled from 3 independent experiments, error bars are SEM. * = p<0.001. (TIFF) [file pone.0074659.s001.tif]

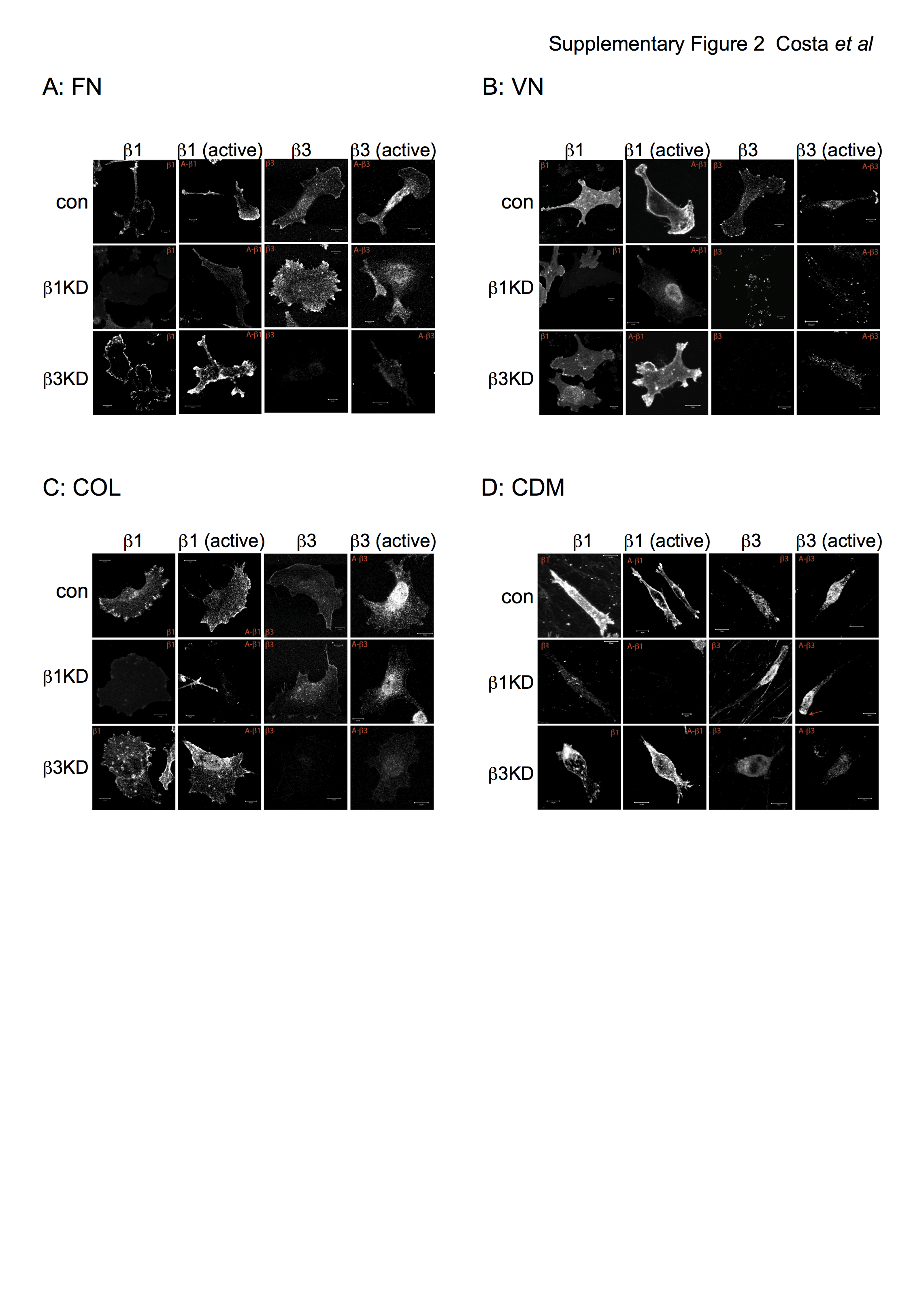

Supplement: Figure S2 — Altered integrin activation in knockdown cells. (A) Example confocal images of cells plated on fibronectin (FN), fixed and stained for specified integrins (either active or total). (B) As in (A) but cells plated on vitronectin (VN). (C) As in (A) but cells plated on Collagen I (COLI). (D) As in (A) but cells plated in cell-derived matrices (CDM). Scale bars 10 µm. (TIFF) [file pone.0074659.s002.tif]

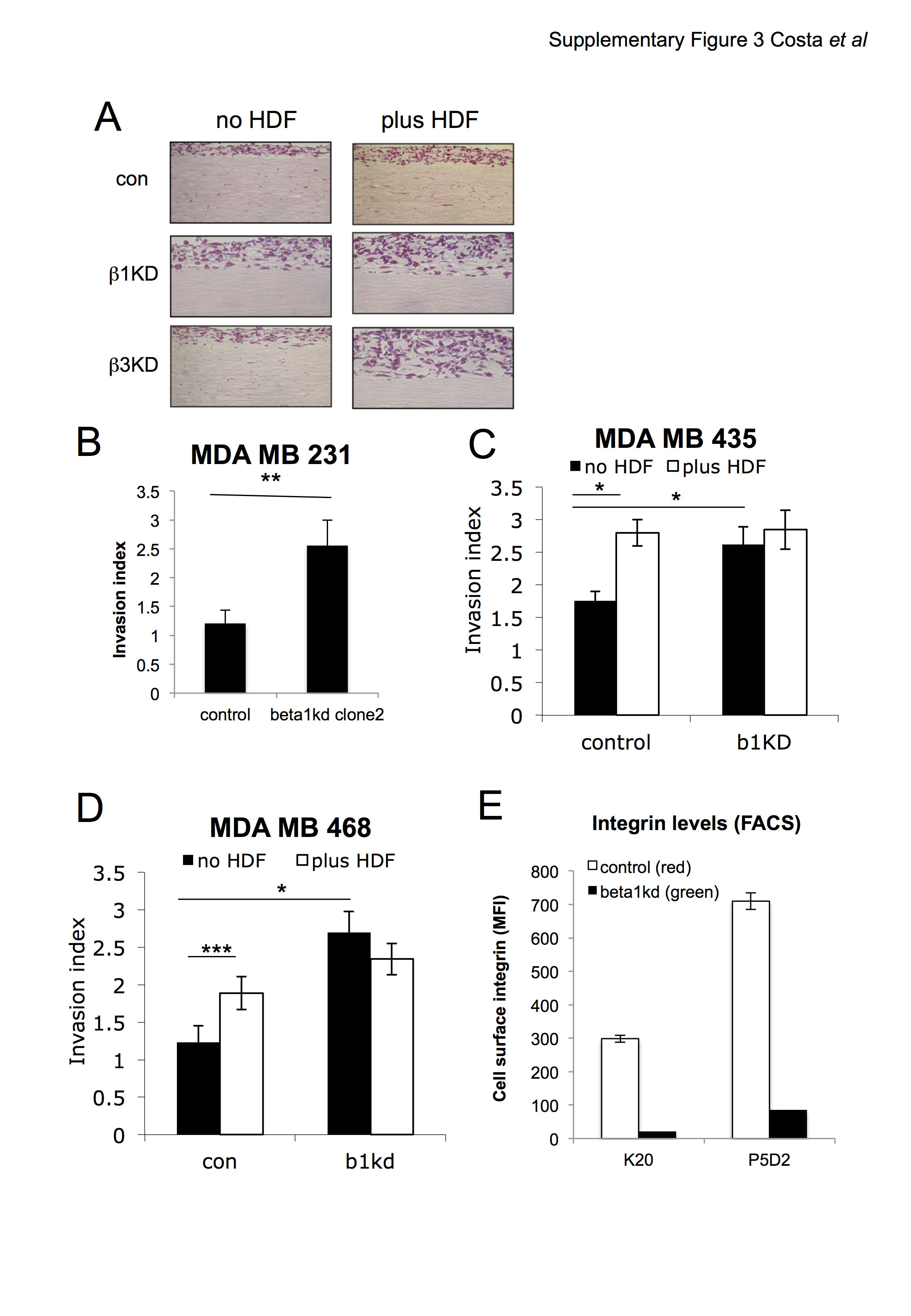

Supplement: Figure S3 — Integrin dependent invasion of human breast carcinoma cells. (A) Example H&E stained sections of organotypic cultures using MDA MB 231 cells with or without fibroblasts. (B) cells expressing control shRNA or β1-specific shRNA (clone#2) in the absence of fibroblasts. (C, D) Quantification of cell invasion in 3D ECM organotypic model using MDA MB 435 (C) or MDA MB 468 (D) in the presence or absence of fibroblasts (HDF). (E) FACS analysis of β1-integrin levels on control and knockdown cells from the same experiments used for injection in mice for analysis of lung extravasation (Figure 2C). Bars are mean +/−SEM pooled from at least two independent experiments, each performed in triplicate. (TIFF) [file pone.0074659.s003.tif]

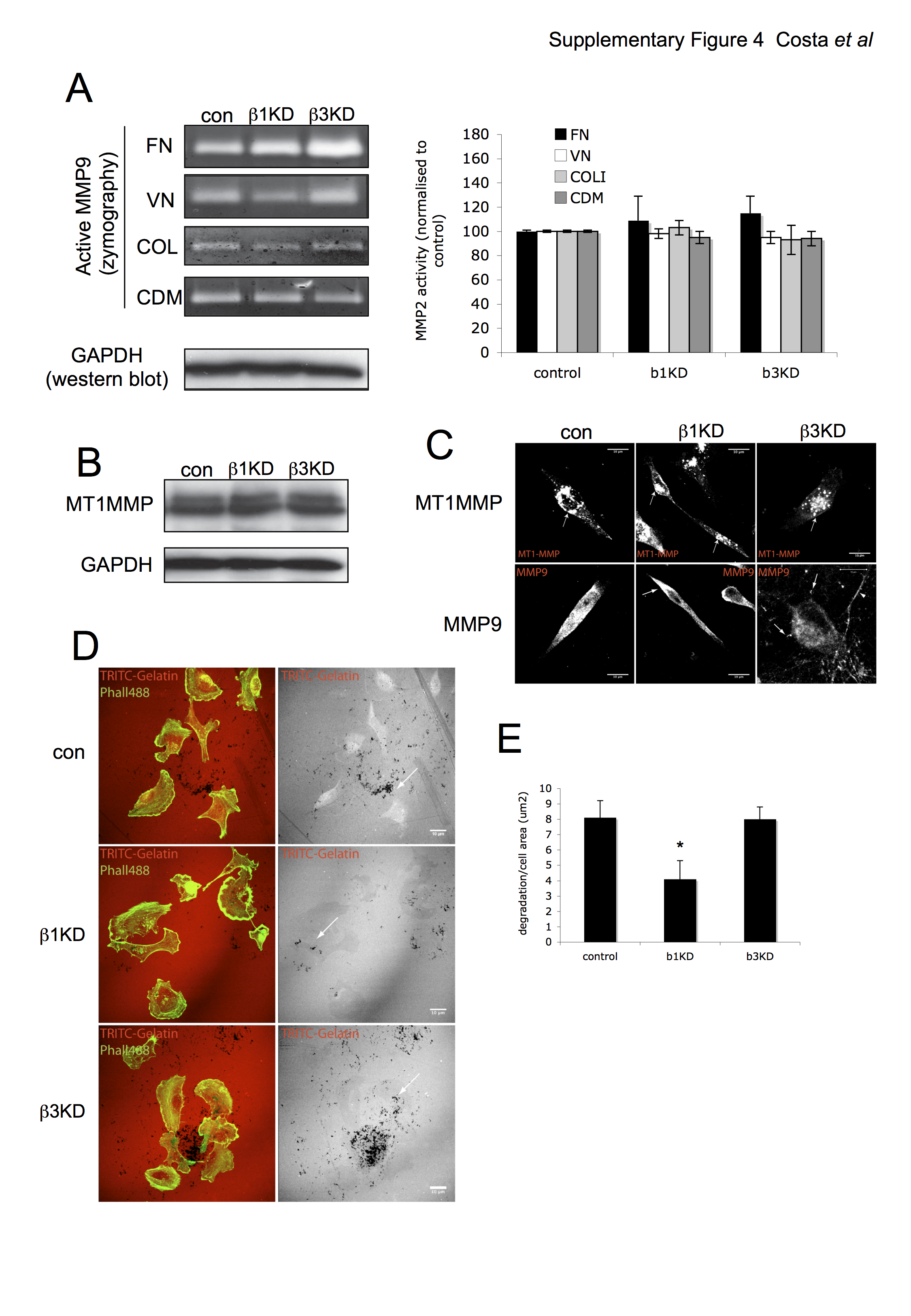

Supplement: Figure S4 — Integrin knockdown results in increased cell protrusion in 3D matrices. (A)Example projected images of >10 confocal z-slices of control or β1 integrin (clone#2) knockdown cells expressing GFP-lifeact. Scale bars are 10 µm. (B) Quantification of protrusion area as a function of total cell area calculated from images as in Fig 4. Bars represent mean % protrusion area per cell +/−SEM from 30 cells over 3 independent experiments. * = p<0.01. (TIFF) [file pone.0074659.s004.tif]

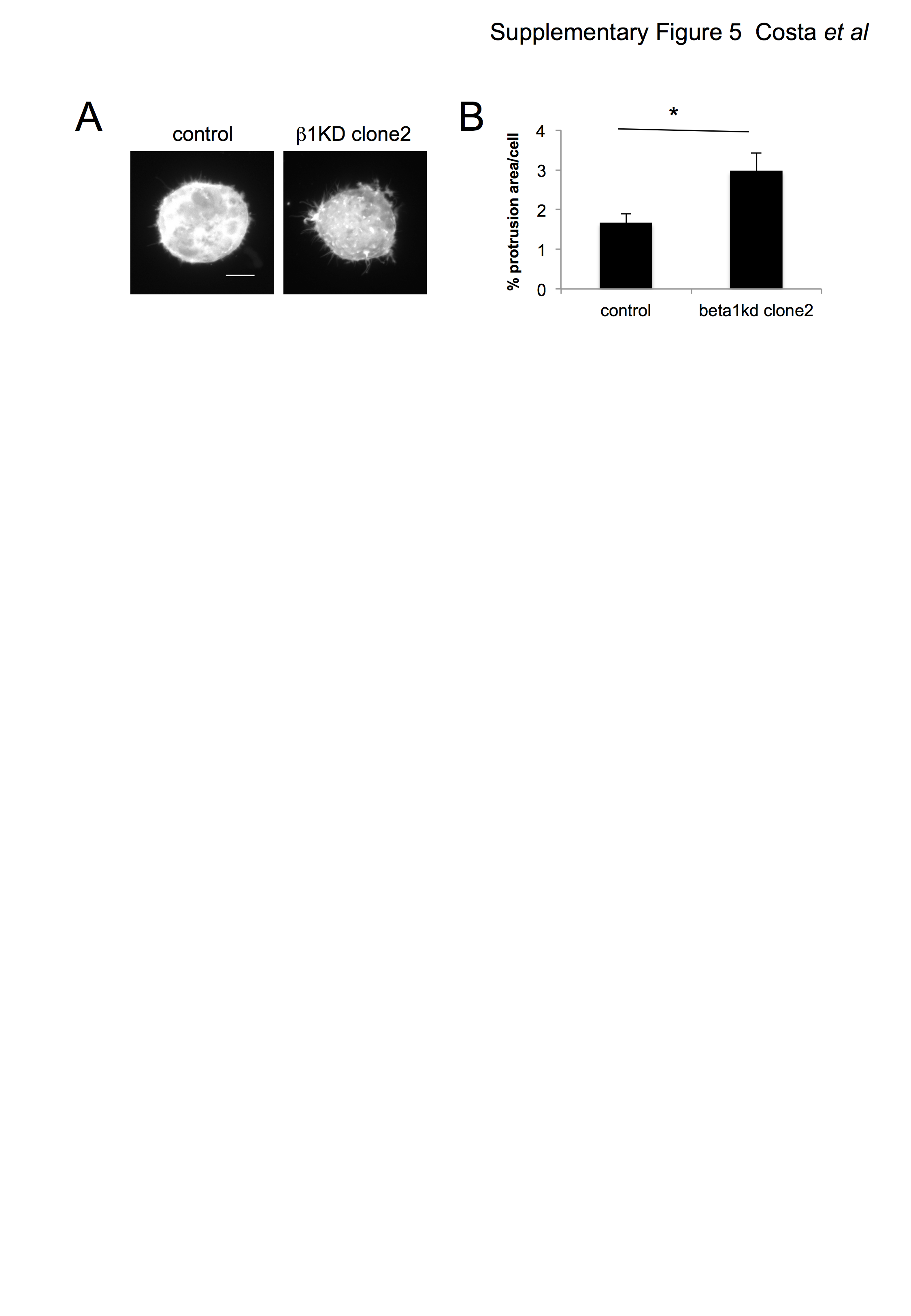

Supplement: Figure S5 — Silencing β1 integrins does not alter MMP levels or activation but decreases 2D gelatin degradation. (A) Example images of lysates from each cell line analysed on zymography gels for active MMP9 levels. Western blot of GAPDH also shown as loading control. Graph shows quantification of active MMP9 levels from zymography experiments. Values are from densitometry analysis from 4 independent experiments for each normalised for loading (from western blot analysis of total MMP9 for each experiment). Bars are mean+/− SEM. (B) Western blots analysis of total MT1-MMP levels in cell lysates. GAPDH is a loading control (C) Example confocal images of cells plated in cell-derived matrices and stained for MT1MMP or MMP9. Arrows show localised recruitment of MMP. (D) Example images from gelatin degradation assay. Cells plated on TRITC-gelatin (red, left panels, black and white in right panels), fixed and stained with phalloidin-Alexa488 (green). Arrows show area of gelatin degradation seen as black dots. (E) Quantification of degradation area normalised for total cell area (presented as µm2). Bars are average area +/−SEM. * = p<0.01 compared to control. (TIFF) [file pone.0074659.s005.tif]

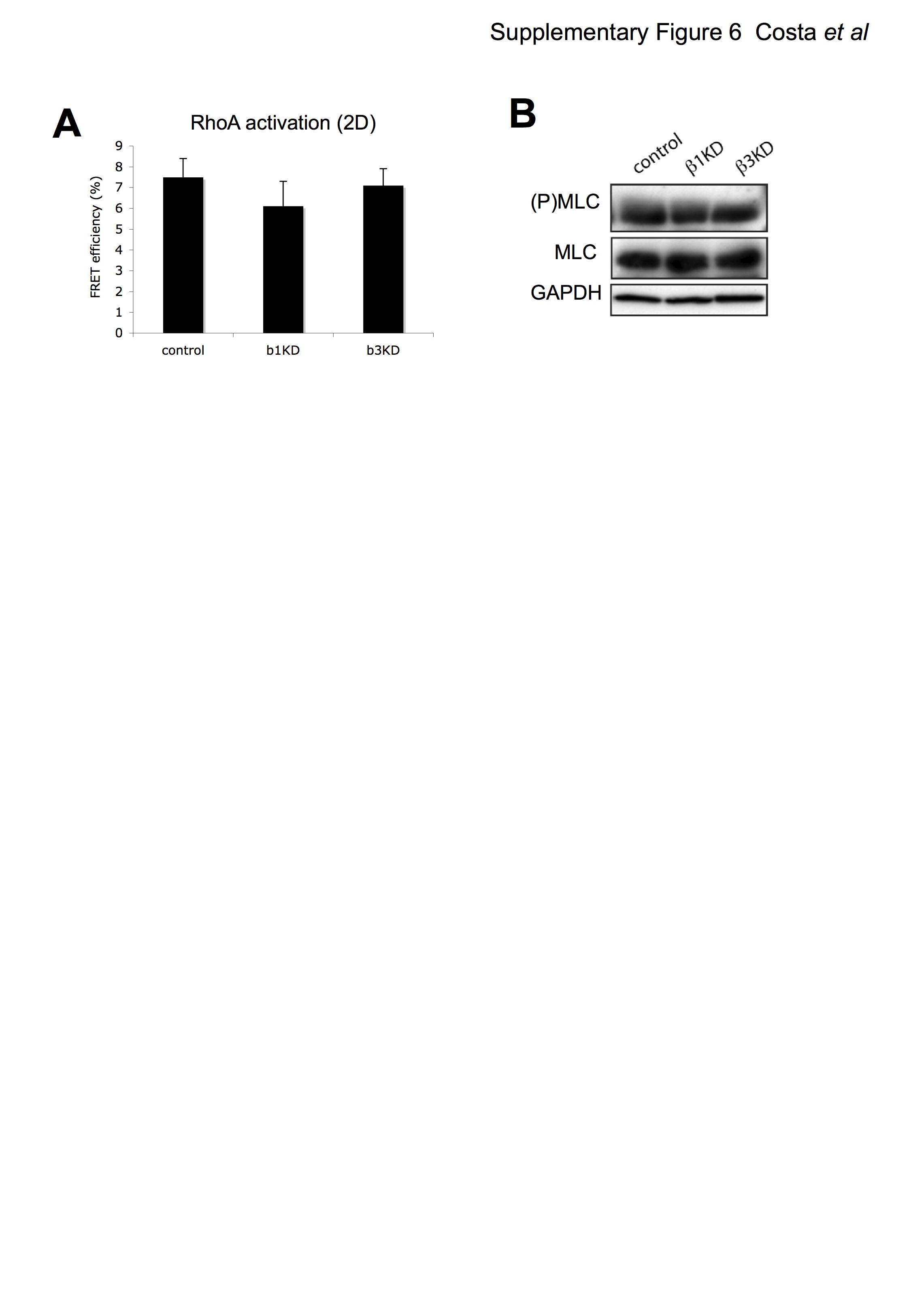

Supplement: Figure S6 — RhoA activation in 3D gels is integrin-dependent. (A) Quantification of RhoA activation using analysis of RhoA FRET in cells plated on 2D glass coverslips. Bars are mean FRET efficiency +/−SEM, n = 18 cells over 3 independent experiments. (B) Representative blots of lysates from control or integrin silenced cells analysed by western blotting for levels of total or phospho (Ser18/Thr19)-myosin light chain (MLC). GAPDH serves as a loading control. Experiment performed 5 times with similar results. (TIFF) [file pone.0074659.s006.tif]

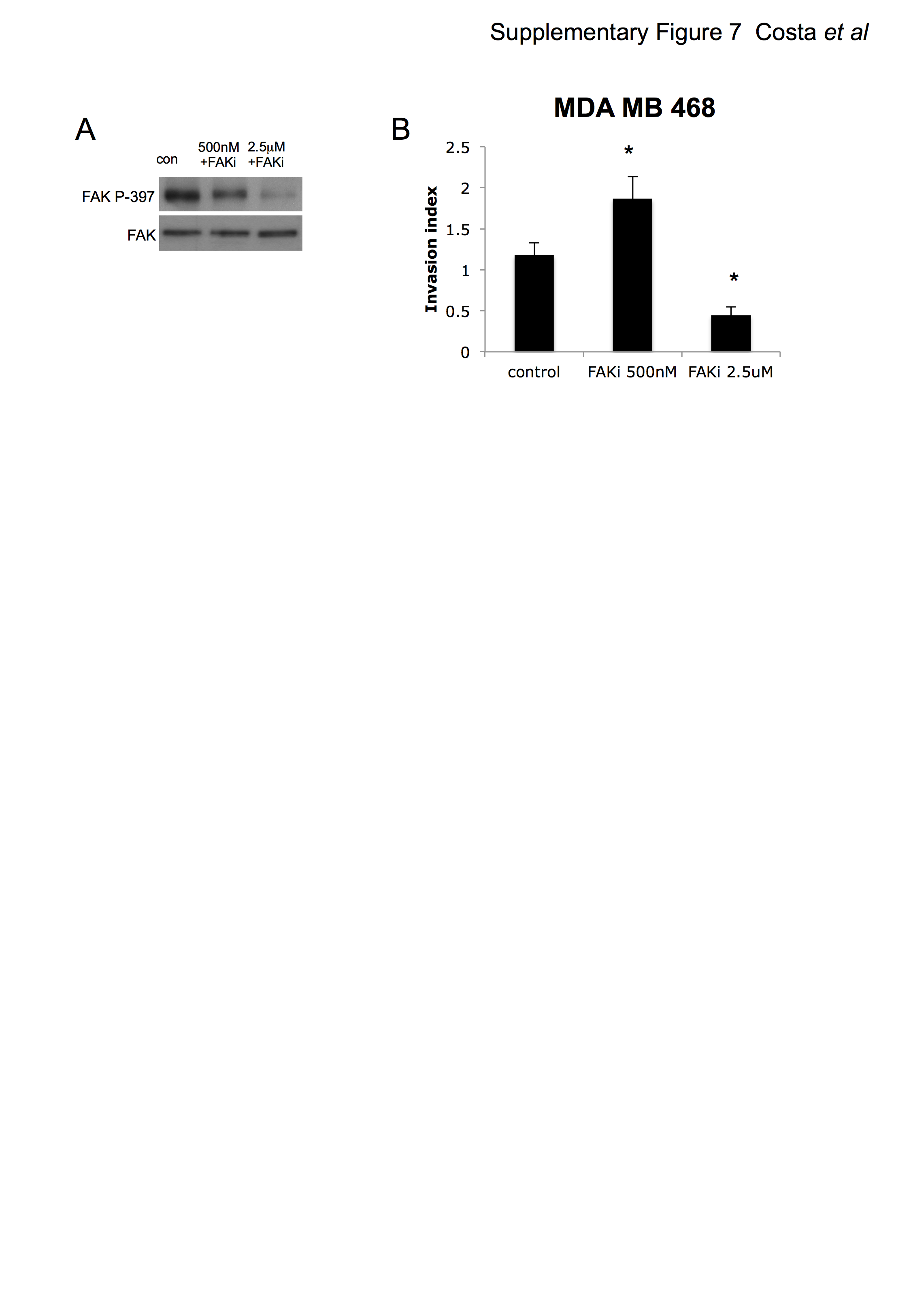

Supplement: Figure S7 — Reduced active FAK leads to increased invasion in MDA MB 468 cells. (A) Western blots of lysates from MDA MB 468 cells treated with vehicle control (con; DMSO), 50nM or 2.5mM of PF-228, lysed and probed for P-Y397 FAK or total FAK. Experiment was performed 3 times with similar results. (B) Quantification of invasion of MDA MB 468 cells into 3D gels treated with DMSO (control) or PF-228 (FAKi) at specified concentrations. Bars represent mean+/−SEM of 18 images across 3 independent experiments. * = p<0.05. (TIFF) [file pone.0074659.s007.tif]
